# Incidence of Skin and Respiratory Immune-Related Adverse Events Correlates With Specific Tumor Types in Patients Treated With Checkpoint Inhibitors

**DOI:** 10.3389/fonc.2020.570752

**Published:** 2021-01-15

**Authors:** Lynn M. Rose, Hannah A. DeBerg, Prakash Vishnu, Jason K. Frankel, Adarsh B. Manjunath, John Paul E. Flores, David M. Aboulafia

**Affiliations:** ^1^ Scientific Administration, Benaroya Research Institute at Virginia Mason, Seattle, WA, United States; ^2^ Systems Immunology, Benaroya Research Institute at Virginia Mason, Seattle, WA, United States; ^3^ Division of Hematology, CHI Franciscan Medical Group, Seattle, WA, United States; ^4^ Section of Urology, Virginia Mason Medical Center, Seattle, WA, United States; ^5^ Department of Hematology and Oncology, Virginia Mason Medical Center, Seattle, WA, United States; ^6^ Division of Hematology, University of Washington, Seattle, WA, United States

**Keywords:** checkpoint inhibitors, immunotherapy, immune-related adverse events, biomarkers, survival benefits, preexisting conditions, autoimmune disease

## Abstract

Checkpoint inhibitors (CPIs) increase antitumor activity by unblocking regulators of the immune response. This action can provoke a wide range of immunologic and inflammatory side effects, some of which can be fatal. Recent studies suggest that CPI-induced immune-related adverse events (irAEs) may predict survival and response. However, little is known about the mechanisms of this association. This study was undertaken to evaluate the influence of tumor diagnosis and preexisting clinical factors on the types of irAEs experienced by cancer patients treated with CPIs. The correlation between irAEs and overall survival (OS) was also assessed. All cancer patients treated with atezolizumab (ATEZO), ipilimumab (IPI), nivolumab (NIVO), or pembrolizumab (PEMBRO) at Virginia Mason Medical Center between 2011 and 2019 were evaluated. irAEs were graded according to the Common Terminology Criteria for Adverse Events (Version 5) and verified independently. Statistical analyses were performed to assess associations between irAEs, pre-treatment factors, and OS. Of the 288 patients evaluated, 59% developed irAEs of any grade, and 19% developed irAEs of grade 3 or 4. A time-dependent survival analysis demonstrated a clear association between the occurrence of irAEs and OS (*P* < 0.001). A 6-week landmark analysis adjusted for body mass index confirmed an association between irAEs and OS in non-Small Cell Lung Cancer (NSCLC) (*P* < 0.03). An association between melanoma and skin irAEs (*P* < 0.01) and between NSCLC and respiratory irAEs (*P* = 0.03) was observed, independent of CPI administered. Patients with preexisting autoimmune disease experienced a higher incidence of severe irAEs (*P* = 0.01), but not a higher overall incidence of irAEs (*P* = 0.6). A significant association between irAEs and OS was observed in this diverse patient population. No correlation was observed between preexisting comorbid conditions and the type of irAE observed. However, a correlation between skin-related irAEs and melanoma and between respiratory irAEs and NSCLC was observed, suggesting that many irAEs are driven by a specific response to the primary tumor. In patients with NSCLC, the respiratory irAEs were associated with a survival benefit.

## Introduction

Immune checkpoint inhibitors (CPIs) that target the cytotoxic T-lymphocyte-associated antigen 4 (CTLA-4) and the programmed cell death protein-1 (PD-1)/programmed cell death ligand-1 (PD-L1) pathway have demonstrated remarkable efficacy across a wide variety of cancer types (1). In addition to inducing durable anti-tumor responses and improving survival of patients, treatment with CPIs is often better tolerated than conventional cytotoxic chemotherapeutic agents. However, CPIs can lead to immune-related adverse events (irAEs) that result in significant morbidity and discontinuation of therapy (2). Recent studies suggest that CPI-induced irAEs can predict survival and response, however little is known about the mechanisms of this association (3, 4).. We undertook this study to evaluate the relationship between tumor type, preexisting clinical factors, the occurrence and type of irAEs, and OS in a diverse set of cancer patients treated with CPIs. Accurately measuring the prognostic significance of preexisting clinical factors could help identify those patients at higher or lower risk for developing irAEs (5–8).

## Materials and Methods

### Patients

This retrospective study included all cancer patients treated at the Virginia Mason Medical Center (Seattle, WA) between July 2011 and October 2019 with *atezolizumab* (ATEZO)*, ipilimumab* (IPI)*, nivolumab* (NIVO), or *pembrolizumab* (PEMBRO). No exclusions were based on preexisting conditions or other clinical factors that frequently preclude participation in clinical trials. Thus, these data represent “real world” patients and treatment responses (9). Patients were only excluded if they were on a blinded clinical trial where their CPI was unknown or if there was missing clinical data. Institutional review board approval was obtained prior to initiation of the study. All data were gathered retrospectively (by LR and AM) using diagnostic codes, search terms, and manual chart review.

### Clinical Measures

Patients were separated into three groups: those treated with inhibitors of CTLA-4 (IPI) alone, CTLA-4 in combination with PD-1 (IPI and NIVO), or PD-1/PD-L1 inhibitors alone (ATEZO, NIVO, or PEMBRO). Data collection included patient demographics (e.g., gender, age, and body mass index [BMI]), primary diagnoses, dates of CPI administration and pre-infusion laboratory test results. All irAEs were graded 1–5 according to the Common Terminology Criteria for Adverse Events (Version 5) (10) and verified independently by the treating physicians. No grade 5 irAEs were recorded. Grade 4 irAEs were life-threatening, requiring urgent hospitalization and suspension of CPI treatment. Preexisting diseases were grouped as: cardiovascular (e.g., coronary artery disease), endocrine/metabolic (e.g., type 2 diabetes mellitus), allergy and/or asthma, autoimmune disease, gastrointestinal (e.g., GERD, colitis), and prior cancers unrelated to the CPI-treated diagnosis. Pre-treatment laboratory test results were categorized according to whether the reported result fell within, above, or below the normal ranges. Prior treatment with steroids (e.g., dexamethasone) within the six months prior to checkpoint inhibitor therapy was also assessed as a possible mitigator of irAEs and overall survival. For purposes of our study, we defined sustained steroid use to be the administration of a prednisone-equivalent dose of 20 mg or higher at least four times in the six months prior to CPI administration

### Statistical Analysis

We calculated summary statistics for demographic and clinical variables and for subgroups categorized by CPI treatment regimen. A Chi-square or Fisher’s exact test was used to evaluate relationships between categorical variables, including cancer diagnosis, preexisting disease, demographic variables, and the grade of the irAE. For patients who were treated with more than one CPI regimen, irAEs associated with the first CPI were used for analysis to avoid introducing bias from double-counting these individuals. Logistic regression was used to model the odds of experiencing an irAE within the first 6 weeks of treatment. Two statistical methods were used to assess the association between irAEs and OS. In the first, a time-dependent Cox proportional hazard regression was performed. In this analysis, irAE experience was modeled as a binary time-dependent covariate for the entire study population. In the second, a landmark approach to Cox proportional hazards regression with a landmark time of 6 weeks was employed to assess OS models (11). Patients with survival times of less than 6 weeks after first CPI treatment (n = 36) were excluded from the both the irAE development and landmark survival analyses. Patients with incomplete irAE records (n = 6) were also excluded. Patients who were treated in an adjuvant setting (n = 20) were excluded from the survival analysis. A heat map showing the incidence of irAEs according to cancer diagnosis was generated using a Fisher’s exact test. A false discovery rate multiple testing correction was applied to the test.

## Results

### Patient Demographics

A total of 321 CPI treatment courses were identified, corresponding to 288 individual patients (**Tables 1** and **2**). Patients were predominantly men (62%) with a median age of 69 years. Almost all patients had metastatic disease (94%). The most common diagnoses were non-Small Cell Lung Cancer (NSCLC-31%), melanoma (24%), urothelial cancer (13%), and renal cell carcinoma (9%) (**Table 1**). The distribution of treatments over time is provided in **Table 2**. Comorbid conditions were grouped as: cardiovascular (n=204); endocrine/metabolic (n = 99); allergy and/or asthma (n = 64), autoimmune disease (n = 58), gastrointestinal (n = 127) and prior cancers unrelated to the CPI-treated diagnosis (n = 96). The cases of preexisting autoimmune disease included: autoimmune thyroid disease (n = 17), rheumatoid arthritis (n = 9), Crohn’s disease or ulcerative colitis (n = 9), systemic lupus erythematosus (n = 3), psoriasis (n = 5), polymyalgia rheumatica (n=3), multiple sclerosis (n = 2), Raynaud’s syndrome (n = 2), chronic lichen planus (n = 2), and ankylosing spondylitis (n = 2). Five individuals had more than one autoimmune disease.

**Table 1:** Patient cohort demographics.

	CTLA-4	CTLA-4/PD-1	PD-1/PD-L1	Overall
**Number**	37	18	233	288
**Age, median (range)**	68 (25-90)	68 (44–79)	70 (30–95)	69.5 (25–95)
**Body mass index median (range)**	29 (19–52)	28 (18–38)	25 (16–46)	25 (16–52)
**Sex, n (%)**				
Male	27 (73)	14 (78)	138 (59)	179 (62)
Female	10 (27)	4 (22)	95 (41)	109 (38)
**Adjuvant therapy, n (%)**	9 (24)	0 (0)	11 (5)	20 (7)
**Metastatic disease, n (%)**	37 (100)	17 (94)	216 (93)	270 (94)
**Cancer diagnosis, n (%)**				
Bladder	0 (0)	0 (0)	37 (16)	37 (13)
Melanoma	36 (97)	5 (28)	27 (12)	68 (24)
NSCLC	0 (0)	0 (0)	89 (38)	89 (31)
RCC	0 (0)	11 (61)	14 (6)	25 (9)
Other^a^	1 (3)	2 (11)	66 (28)	69 (24)
**Preexisting conditions, n (%)**				
Cardiovascular	22 (59)	14 (78)	168 (72)	204 (71)
Gastrointestinal	13 (35)	8 (44)	106 (45)	127 (44)
Endocrine or metabolic	7 (19)	4 (22)	88 (38)	99 (34)
Other cancer	10 (27)	3 (17)	83 (36)	96 (33)
Allergy or asthma	7 (19)	6 (33)	51 (22)	64 (22)
Allergy	6 (16)	3 (17)	38 (16)	47 (16)
Asthma	3 (8)	3 (17)	21 (9)	27 (9)
Autoimmune disease	6 (16)	4 (22)	48 (21)	58 (20)

^a^Head and neck (n = 16), colorectal (n = 7), hepatocellular (n = 7), esophageal (n = 7), and other (n = 32).

**Table 2:** CPI use over time. Number of CPI treatment courses administered by year.

	2011	2012	2013	2014	2015	2016	2017	2018	2019^a^	Total
**CTLA-4**	5	7	8	9	7	2	0	1	0	39
**CTLA-4/PD-1**	0	0	0	0	2	3	4	8	6	23
**PD-1/PD-L1**	0	0	0	1	11	56	60	78	53	259
**Total**	5	7	8	10	20	61	64	87	59	321

^a^Data collected from July 2011 through October 2019.

### Overall Incidence of irAEs

**Figure 1:**
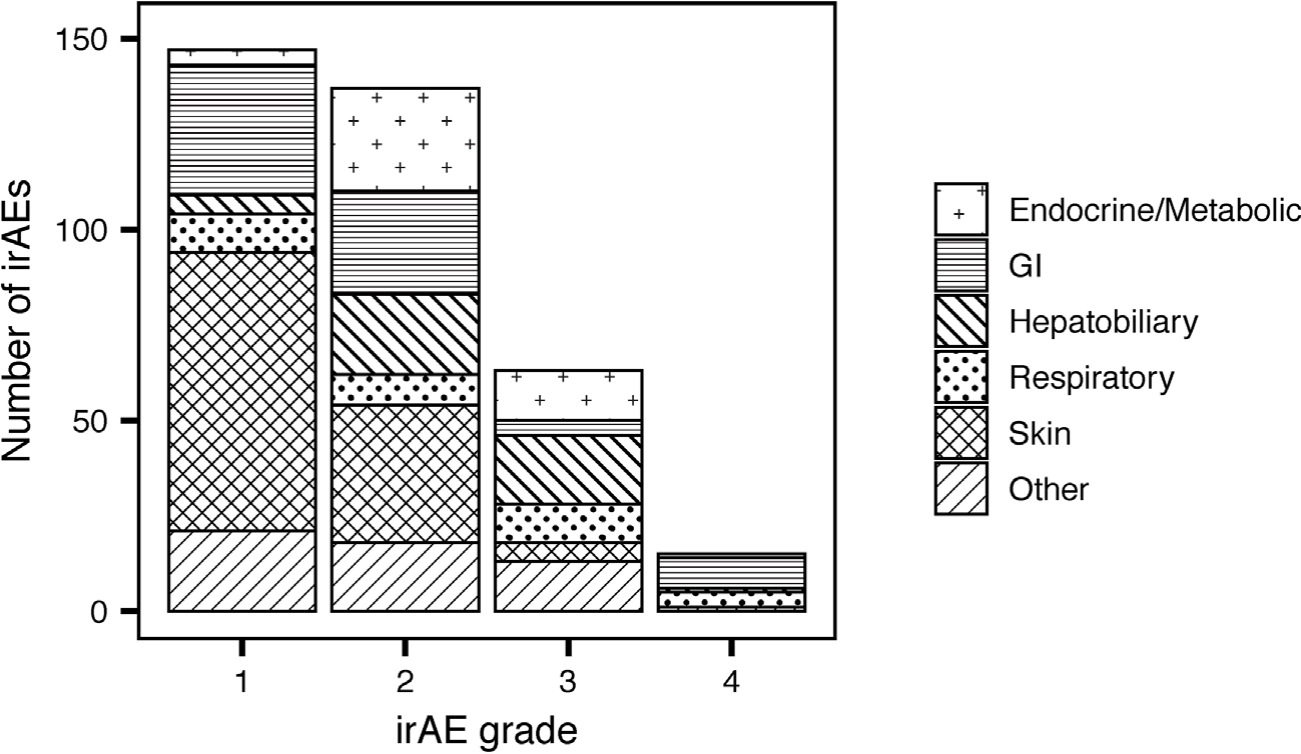
Numbers of reported immune-related adverse events (irAEs) by severity and type.

Fifty-nine percent of patients treated with CPIs (**Table 3**) developed irAEs (n = 171). Severe irAEs (grade 3 or 4) occurred in 29% of patients (n = 55) (**Table 3**). The median time to first irAE was 4 weeks. The median time from initial CPI treatment to the most severe irAE was 10 weeks. Of the irAEs evaluated, 147 (41%) were grade 1, 137 (38%) grade 2, 63 (18%) grade 3, and 15 (4%) grade 4. Maculopapular skin rashes and pruritis were the most frequently reported irAEs with 34% of patients reporting at least one skin-related irAE. Other common irAEs were immune-mediated colitis and other gastrointestinal irAEs (23% of patients), endocrine or metabolic adverse events (14%), and respiratory irAEs (11%) (e.g., pneumonitis). Hepatobiliary, blood, eye, musculoskeletal, nervous system, and renal irAEs each affected less than 10% of patients. The grade 3 and 4 irAEs were predominantly respiratory, hepatobiliary, gastrointestinal, and endocrine. One patient developed chronic type 1 diabetes mellitus because of treatment with PEMBRO. **Figure 1** presents the distribution of irAEs by severity and type.

**Table 3:** irAE incidence. Numbers and percentages of patients who experienced at least one irAE of the specified type. Numbers inside of parentheses indicate percentages of patients relative to the CPI treatment group.

irAE Type	CTLA-4 (n = 37)			CTLA-4/PD-1 (n = 18)			PD-1/PD-L1 (n = 233)			Overall (n = 288)		
	All	Grades 1–2	Grades 3–4	All	Grades 1–2	Grades 3–4	All	Grades 1–2	Grades 3–4	All	Grades 1–2	Grades 3–4
Blood	0 (0)	0 (0)	0 (0)	1 (6)	1 (6)	0 (0)	1 (0)	0 (0)	1 (0)	2 (1)	1 (0)	1 (1)
Cardiac	0 (0)	0 (0)	0 (0)	0 (0)	0 (0)	0 (0)	1 (0)	0 (0)	1 (0)	1 (0)	0 (0)	1 (1)
Dermatological	18 (49)	16 (43)	2 (5)	12 (67)	12 (67)	0 (0)	62 (27)	59 (25)	3 (1)	102 (32)	97 (39)	5 (7)
Endocrine/ Metabolic	5 (14)	2 (5)	4 (11)	2 (11)	1 (6)	1 (6)	29 (12)	24 (10)	5 (2)	39 (12)	28 (11)	11 (16)
Eye	0 (0)	0 (0)	0 (0)	1 (6)	0 (0)	1 (6)	5 (2)	5 (2)	0 (0)	6 (2)	5 (2)	1 (1)
Gastrointestinal	14 (38)	10 (27)	4 (11)	9 (50)	7 (39)	3 (17)	40 (17)	36 (15)	4 (2)	66 (21)	55 (22)	11 (16)
Hepatobiliary	4 (11)	2 (5)	2 (5)	5 (28)	2 (11)	5 (28)	15 (6)	11 (5)	6 (3)	39 (12)	20 (8)	19 (27)
Musculo-skeletal	2 (5)	2 (5)	0 (0)	1 (6)	0 (0)	1 (6)	18 (8)	16 (7)	3 (1)	22 (7)	18 (7)	4 (6)
Nervous system	0 (0)	0 (0)	0 (0)	2 (11)	2 (11)	0 (0)	10 (4)	8 (3)	2 (1)	14 (4)	11 (4)	3 (4)
Renal	0 (0)	0 (0)	0 (0)	0 (0)	0 (0)	0 (0)	2 (1)	1 (0)	1 (0)	2 (1)	1 (0)	1 (1)
Respiratory	1 (3)	1 (3)	0 (0)	1 (6)	1 (6)	0 (0)	26 (11)	13 (6)	13 (6)	28 (9)	15 (6)	13 (19)

### Factors Impacting irAE Development

**Figure 2:**
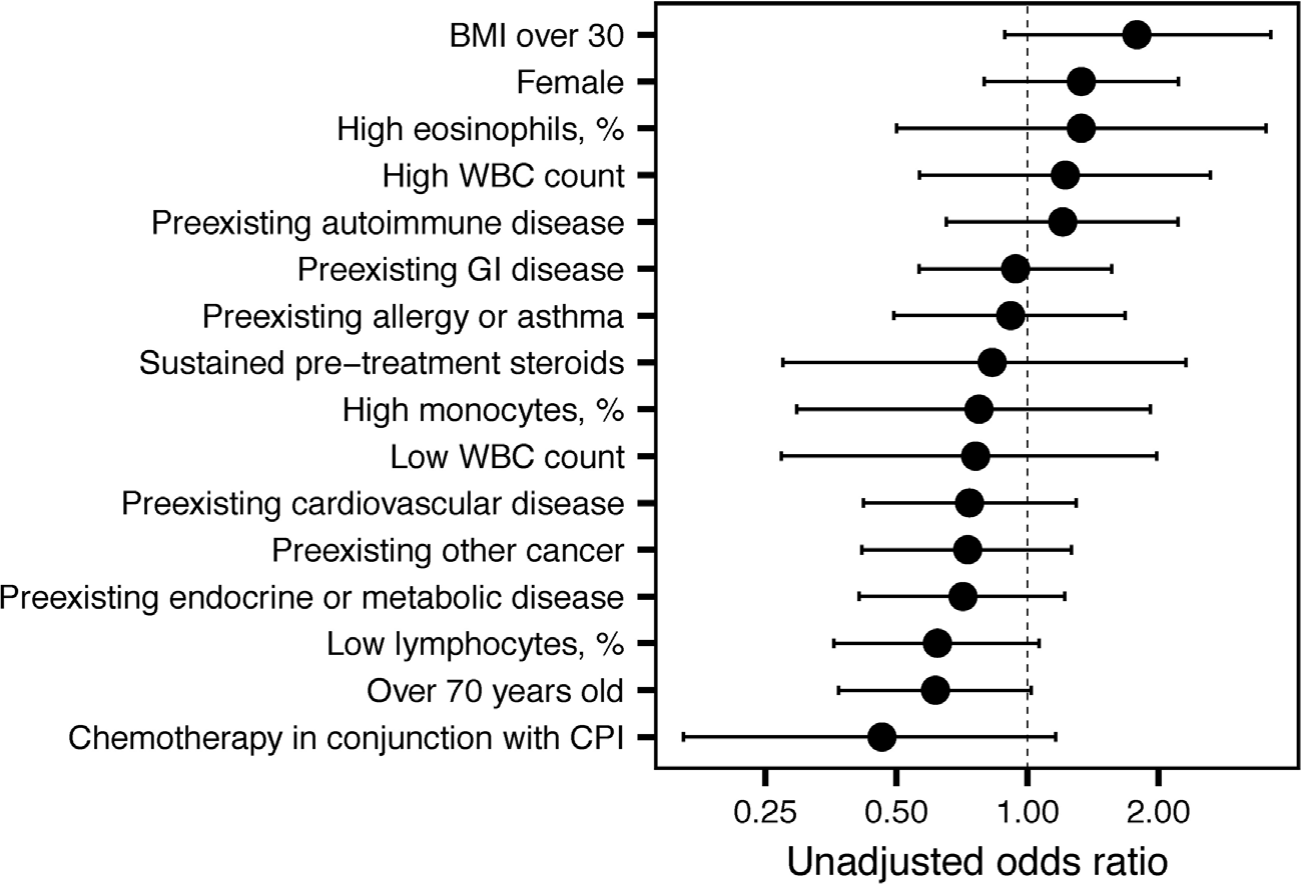
Unadjusted odds ratios for immune-related adverse event (irAE) development. Unadjusted odds ratios and 95% confidence intervals are shown for the indicated variables.

Anti-PD-1/PD-L1-treated patients were less likely to experience an irAE than patients treated with anti-CTLA-4 (OR=0.33, 95% CI 0.15 to 0.70, *P* = 0.004). Odds ratios (OR) for the occurrence of irAEs are shown in **Figure 2**.** A** 6-week landmark analysis was applied to pre-treatment OR calculations to avoid potential bias from patients with very short survival times. No other factors were significantly associated with increased odds of irAE development (**Figure 2**). As previously reported, two factors decreased odds of irAE development, age > 70 and low pre-infusion lymphocyte counts (*P* = 0.05) (5, 6). In subjects who survived for at least 6 weeks, there was an association between preexisting autoimmune disease and the occurrence of a severe irAE (grade 3 or 4; *P* = 0.01, Chi-squared test).

### Categories of irAEs

**Figure 3:**
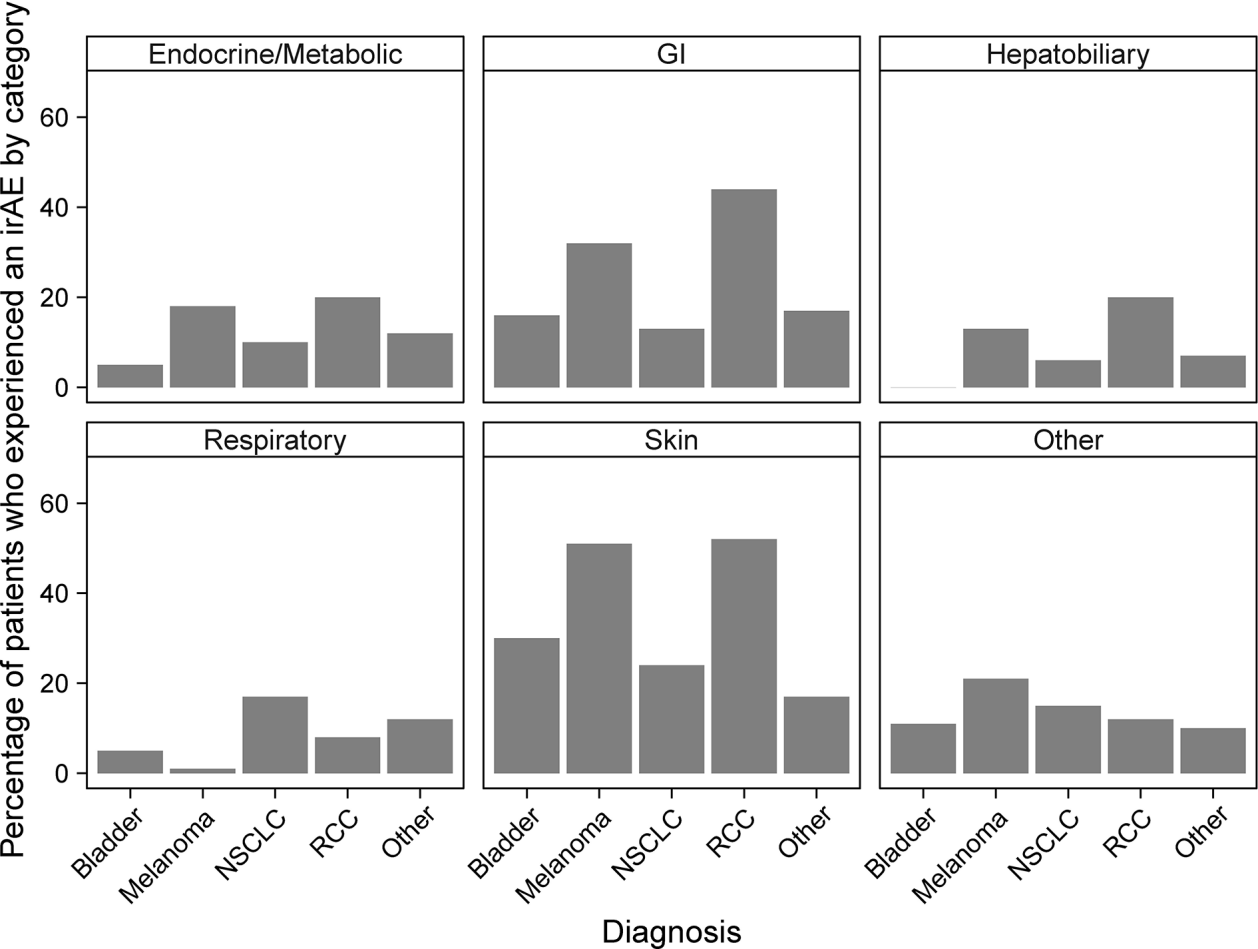
Percentages of patients who experience immune-related adverse events (irAEs) by cancer diagnosis and irAE type. Bars report the percentage of patients with the indicated diagnosis who experienced at least one irAE of the type indicated by the panel title.

**Figure 4:**
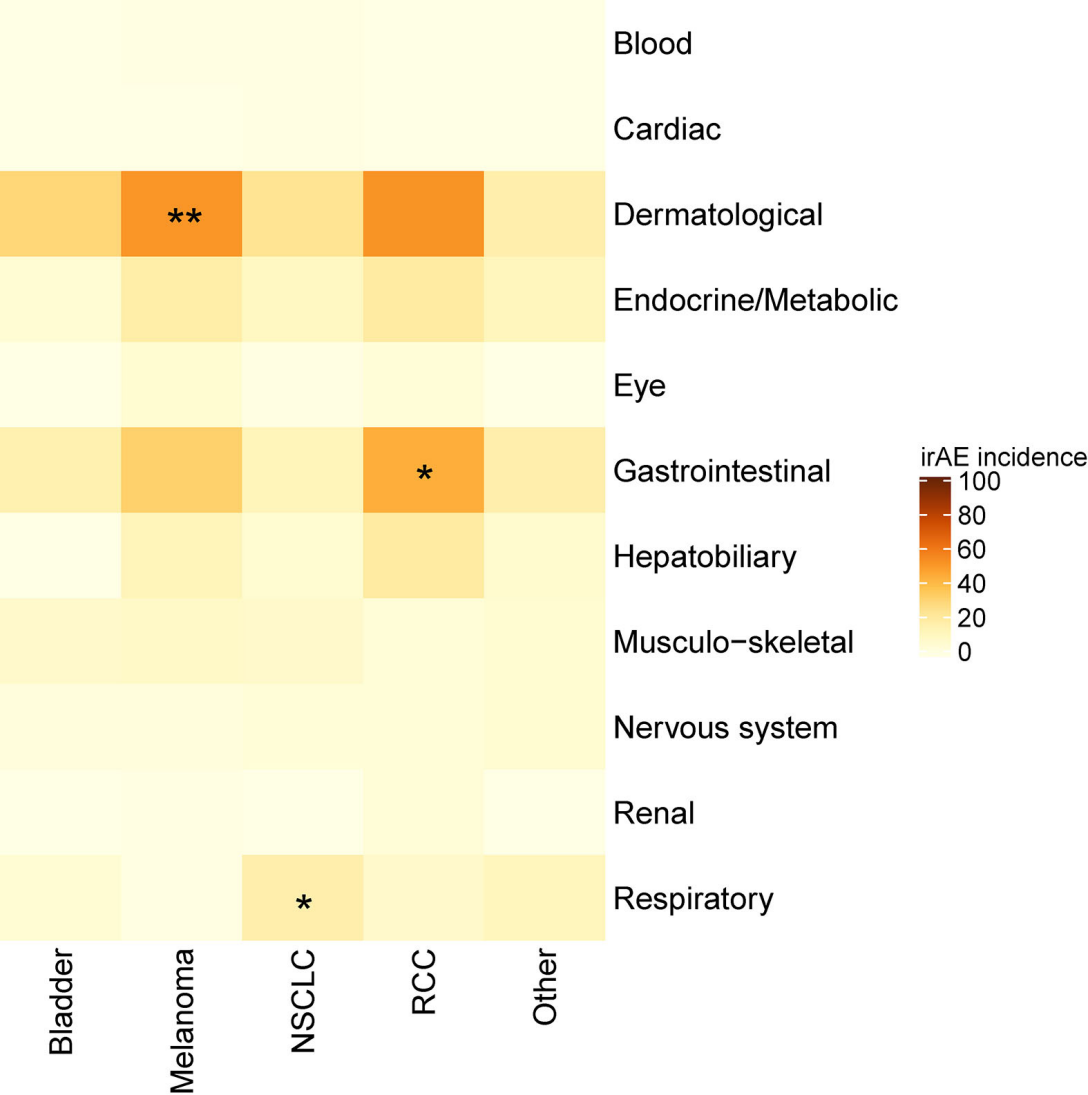
Heatmap showing the incidence of immune-related adverse events (irAEs) according to cancer diagnosis. Color indicates the percent of individuals with a diagnosis (column) who experienced an irAE of the indicated class (row). Asterisks indicate a significance, *p < 0.05, **p < 0.01 (Fisher’s exact test with a false discovery rate multiple testing correction applied).

An increased incidence of skin-related irAEs was observed in melanoma patients receiving CPI treatment relative to other cancer diagnoses, with 51% of melanoma patients experiencing at least one skin-related irAE. An increased incidence of respiratory irAEs was also observed in patients undergoing CPI therapy for NSCLC with at least one respiratory irAE reported in 17% of NSCLC patients (**Figure 3**). These differences were significant even with a multiple testing correction applied (**Figure 4**). To account for the possible confounding effect of *ipilimumab*, used predominantly in treatment of melanoma patients, we conducted a subgroup analysis of patients only treated with anti-PD-1/PD-L1 CPIs. Within this subset, significantly more respiratory irAEs were reported in NSCLC patients than in patients with other diagnoses (OR = 2.4, *P* < 0.03, Fisher’s exact test) and significantly more skin-related irAEs were reported in melanoma patients than in patients with other diagnoses (OR = 2.9, *P* = 0.01, Fisher’s exact test). No melanoma patients developed vitiligo, a skin irAE reported in the literature. A positive correlation (P < 0.05) between gastrointestinal irAEs was observed in patients with renal cell carcinoma (RCC).

### Factors Impacting Survival

**Figure 5:**
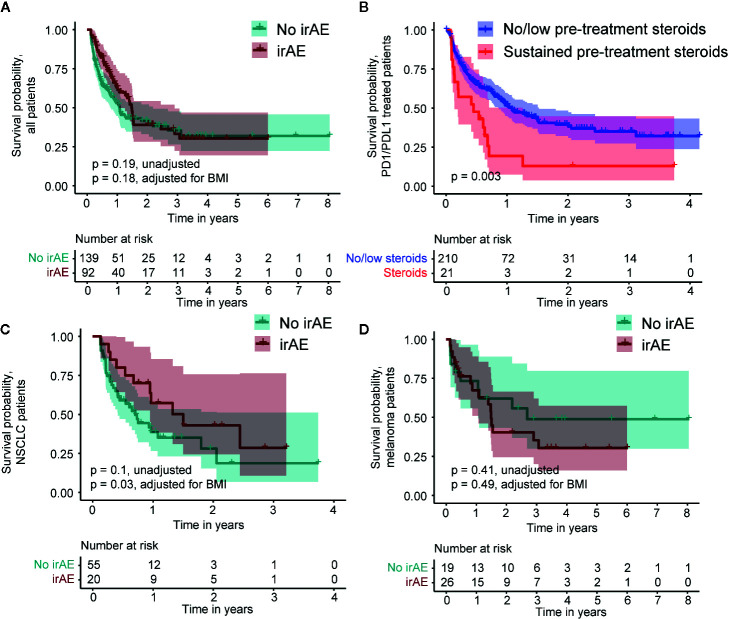
Kaplan-Meier survival curves comparing patients who do and do not experience an immune-related adverse event (irAE) in the 6-week landmark analysis and survival relative to pre-treatment steroid administration. **(A)** Survival of all patients. **(B)** Survival relative to pre-treatment steroid administration in PD1/PDL1 treated patients. **(C)** Survival within the subset of non-Small Cell Lung Cancer (NSCLC) patients. **(D)** Survival within the subset of melanoma patients.

Using a time-dependent Cox proportional hazard regression analysis to model irAEs as a binary time-dependent covariate, we observed a clear relationship between irAE and improved survival (HR = 0.41, 95% CI 0.29 to 0.57, *P* < 0.001) (12–14). To account for potential bias due to variability in survival times, we also employed a landmark approach to assess the effects of irAEs and the impact on OS models (11). We employed a 6-week landmark based on the median time to first irAE and to be consistent with previous studies that have used landmark times between 6 and 12 weeks (3, 12–14). Using this approach, we observed a median survival of 57.1 weeks in patients without irAEs and 78.1 weeks in patients with irAEs. However, these differences were not statistically significant (HR = 0.78, 95% CI 0.54 to 1.13, *P* = 0.19, **Figure 5A**). We observed significantly worse OS in patients who experienced sustained steroid treatment prior to CPI treatment, which may have resulted in a less robust response to CPI. Among PD1/PDL1 treated patients, we identified 21 patients (9%) who received steroids with a prednisone-equivalent dose of 20 mg or higher at least four times in the six months prior to CPI administration. This sustained steroid treatment was associated with significantly worse OS in this patient population (HR = 2.16, 95% CI 1.39 to 3.61, *P* = 0.003, **Figure 5B**). No patients with sustained steroid treatment were identified in other CPI classes. We also identified 20 individuals who were administered a prednisone-equivalent dose of 20 mg or higher in the month following the first CPI infusion. The OS of this patient group did not differ significantly from the group that did not receive steroids following CPI administration (HR = 1.38, 95% CI 0.33 to 1.56, *P* = 0.41). The only other factor that showed a significant association with OS was pre-treatment BMI > 30 kg/m^2^ (p = 0.03). A subset analysis employing an adjustment for patient BMI of the two largest patient populations, NSCLC and melanoma, revealed a significant OS benefit in patients with NSCLC who experienced irAEs (adjusted HR = 0.43, 95% CI 0.21 to 0.93, *P* = 0.03, **Figure 5C**), but not in melanoma (adjusted HR = 1.35, 95% CI 0.58 to 3.14, *P* = 0.49, **Figure 5D**).

## Discussion

We undertook this study to assess the impact of tumor type and other preexisting factors on the development and type of irAEs, while also examining the effect of irAEs on OS. The study is based on a retrospective analysis of the irAEs observed across a diverse set of malignancies. In this study, no exclusions were imposed based on preexisting conditions or other comorbid conditions that frequently preclude participation in clinical trials, which represent the “real world” experience of the irAEs in cancer patients treated with CPIs and the outcomes.

We employed two methods to evaluate the OS benefit: a landmark survival analysis, which excluded patients who died in the first 6 weeks of treatment and a time-dependent covariate analysis which included all patients. Previous studies of NSCLC and melanoma have reported an association between improved survival and irAE experience using survival analyses with 6 to 12-week landmarks. Our 6-week landmark analysis confirmed an association between survival and irAEs in NSCLC patients but was inadequately powered in the “no-irAE” group to assess survival of melanoma patients on the basis of irAE. The result in NSCLC patients is consistent with previously reported results in which a 6-week landmark analysis of 134 NSCLC patients receiving NIVO demonstrated increased progression-free survival (PFS) and OS in patients who experienced an irAE (3).

The landmark analysis also revealed a trend towards an OS benefit in individuals experiencing an irAE in the first 6 weeks of treatment. For this reason, OS was also calculated as a time-dependent variable which included patients who experienced irAEs within the first 6 weeks of treatment (3, 12–14). This analysis identified a highly significant association between irAEs and OS. Sato and colleagues also noted an association between irAEs and PFS in the absence of a landmark or other accounting for the time-dependence of irAEs in 38 NSCLC patients treated with NIVO (15). Teroaka et al. reported an association between irAEs and survival in NIVO*-*treated NSCLC patients with a 2-week landmark time (16). Based on our comparison of the landmark and time-dependent analyses, we propose that a time-dependent treatment of irAEs may be a more relevant measure of survival benefit, since landmark analyses, by definition, do not account for early irAEs. Relative to time-dependent Cox regression, landmark analyses have been shown to attenuate the effect of a time-dependent covariate and our results are consistent with this observation (11).

None of the preexisting comorbid conditions which we evaluated (e.g., cardiovascular disease, autoimmune disease, etc.) altered the overall incidence or type of irAEs experienced by the various patient subgroups, but the higher incidence of grade 3 or 4 irAEs which we detected in patients with preexisting autoimmune disease did appear to be clinically relevant (*P* = 0.01). However, in this subset of 58 patients, there was no obvious link between the type of irAE experienced and the underlying autoimmune condition. Thus, although exacerbations of preexisting autoimmune disease are a predicted consequence of CPI treatment, this was not observed in our study (17). Our data do suggest that patients with preexisting autoimmune disease are more prone to developing serious irAEs of any type, thus emphasizing the need for careful monitoring of this patient population. Most irAEs were managed with corticosteroids and irAEs subsided without permanent discontinuation of CPI therapy. Recent studies do suggest that limiting the use of corticosteroids to the lowest dose possible to achieve clinical benefit may lead to better control of cancer for these patients (18). In one study, NSCLC patients treated with ≥ 10 mg of prednisone at the time of CPI initiation had worse outcomes than patients who received 0 to < 10 mg of prednisone. The authors suggest that this difference may be driven by a poor-prognosis subgroup of patients who receive corticosteroids for palliative reasons (19).

Our most significant finding is that the lung and skin irAEs correlate with the primary tumor sites for both NSCLC and melanoma. This suggests, that at least in these two diseases, the irAEs are an important biomarker of a specific tumor-directed response. Previous reports have suggested that the incidence of respiratory irAEs is similar between NSCLC and melanoma patients, however our data do not support this conclusion (20, 21). In our cohort of 68 melanoma patients, only one patient experienced pneumonitis, a significantly lower incidence than in NSCLC (p = 0.01, Fisher’s exact test). In this study, the incidence of respiratory irAEs was higher than in other published reports (22). This difference may be due to the challenge in distinguishing pneumonia due to infection from pneumonitis due to CPI treatment. With respect to melanoma, we observed a strong association between the skin irAEs and melanoma, but no association with OS. We hypothesize that the skin rashes, which occur early in the treatment with CPIs, may predict later development of vitiligo, another skin-related irAE that is associated with successful anti-melanoma responses (23). The pathogenesis of vitiligo is unknown, but one potential link may be epitope spreading, in which initial immune activity against one or more tumor-specific epitope extends to antigens shared by melanoma cells and melanocytes (24).

The main limitation of this study is its retrospective design and the inclusion of data generated by multiple treating physicians. Due to potential variability in practice patterns, there could be inconsistencies in the reporting of irAEs, patient follow-up, and administration of interventions to reduce or prevent irAEs. We sought to mitigate these variables by having the treating physicians review their medical records and confirm the irAE grade assignment. Certain irAEs, e.g., pneumonitis, were radiographically diagnosed and therefore not associated with the bias of oncologist’s notation of the irAE. Furthermore, all oncologists involved in this study treated multiple cancer types, which mitigates the risk of reporting diagnosis specific effects being associated with an individual physician. Therefore, we believe that the timing of irAEs post-CPI treatment, was consistent across all treating physicians, suggesting no specific interference of effect.

Use of a broad study interval for CPI experience, in this case July 2011–October 2019, though inclusive for patients, is also a potential limitation given that clinical experience in managing irAEs has increased over time. In addition, the number of CPIs and their applicability across different tumor types have also increased over time. The newer CPIs are purported to have less side effects. A review of treatments by year indicated that the incidence of irAEs remained consistent, although the severity dropped as ipilimumab was replaced by less toxic PD1/PDL1 inhibitors for the treatment of metastatic melanoma. In recent years, the combination of CPIs with cytotoxic chemotherapy (e.g., pembrolizumab plus platinum-based agents), may have contributed to the heterogeneity of reported irAEs. Chemotherapy may have inherent immunosuppressive effects, albeit low and variable, and also some regimens (e.g., carboplatin/paclitaxel) may incorporate a single time low-dose of steroids. These factors may have led to a lower incidence of irAEs in this cohort. With respect to findings of interstitial pneumonia in this cohort, we note that interstitial pneumonitis is a rare complication of paclitaxel and does not increase the incidence of pneumonitis compared to patients treated with pembrolizumab as single therapy (25, 26). While we hope to establish a consistent grading system for future retrospective analyses of responses to therapy, we note that the frequencies of irAEs identified in this study are not dissimilar from those reported in the literature.

In conclusion, there is growing evidence that irAEs may be directly linked to improved survival in cancer patients treated with CPIs. We undertook this retrospective study to identify clinical factors that inhibit or contribute to an active irAE response. A time-dependent analysis confirmed a significant association between irAEs and OS. We observed a clear association between skin-related irAEs and melanoma as well as between respiratory irAEs and NSCLC, suggesting that these two types of irAEs may be driven by specific immune responses to the primary tumor. In patients with NSCLC, the respiratory irAEs were associated with a survival benefit.

## Data Availability Statement

The raw data supporting the conclusions of this article will be made available by the authors, without undue reservation.

## Ethics Statement

The studies involving human participants were reviewed and approved by Benaroya Research Institute Institutional Review Board. Written informed consent for participation was not required for this study in accordance with the national legislation and the institutional requirements.

## Author Contributions

Conception and design: LR, JKF, JPF, PV, and DA. Development of methodology: LR and HD. Acquisition of data: LR and AM. Analysis and interpretation of data: HD and LR. Writing, review, and revision of the manuscript: LR, HD, JKF, JPF, PV, and DA. Administrative, technical, or material support: LR and HD. All authors contributed to the article and approved the submitted version.

## Funding

This work was funded by Benaroya Research Institute, not by a grant.

## Conflict of Interest

The authors declare that the research was conducted in the absence of any commercial or financial relationships that could be construed as a potential conflict of interest.

## References

[1] Keung EZ Wargo JA The current landscape of ommune checkpoint inhibition for solid malignancies. Surg Oncol Clin N Am (2019) 28:369–86. 10.1016/j.soc.2019.02.008 31079794

[2] Schadendorf D Wolchok JD Hodi FS Chiarion-Sileni V Gonzalez R Rutkowski P Efficacy and Safety Outcomes in Patients With Advanced Melanoma Who Discontinued Treatment With Nivolumab and Ipilimumab Because of Adverse Events: A Pooled Analysis of Randomized Phase II and III Trials. J Clin Oncol (2017) 35:3807–14. 10.1200/JCO.2017.73.2289 PMC5791828 28841387

[3] Haratani K Hayashi H Chiba Y Kudo K Yonesaka K Kato R Association of immune-related adverse events with nivolumab efficacy in Non-Small-Cell Lung Cancer. JAMA Oncol (2018) 4:374–8. 10.1001/jamaoncol.2017.2925 PMC6583041 28975219

[4] Rogado J Sanchez-Torres JM Romero-Laorden N Ballesteros AI Pacheco-Barcia V Ramos-Levi A Immune-related adverse events predict the therapeutic efficacy of anti-PD-1 antibodies in cancer patients. Eur J Cancer (2019) 109:21–7. 10.1016/j.ejca.2018.10.014 30682533

[5] Conforti F Pala L Bagnardi V De Pas T Martinetti M Viale G Cancer immunotherapy efficacy and patients’ sex: a systematic review and meta-analysis. Lancet Oncol (2018) 19:737–46. 10.1016/S1470-2045(18)30261-4 29778737

[6] Kugel CH,3 Douglass SM Webster MR Kaur A Liu Q Yin X Age correlates with response to anti-PD1, reflecting age-related differences in intratumoral effector and regulatory T-cell populations. Clin Cancer Res (2018) 24:5347–56. 10.1158/1078-0432.CCR-18-1116 PMC6324578 29898988

[7] McQuade JL Daniel CR Hess KR Mak C Wang DY Rai RR Association of body-mass index and outcomes in patients with metastatic melanoma treated with targeted therapy, immunotherapy, or chemotherapy: a retrospective, multicohort analysis. Lancet Oncol (2018) 19:310–22. 10.1016/S1470-2045(18)30078-0 PMC5840029 29449192

[8] Kennedy LC Bhatia S Thompson JA Grivas P Preexisting autoimmune disease: Implications for immune checkpoint inhibitor therapy in solid tumors. J Natl Compr Canc Netw (2019) 17:750–7. 10.6004/jnccn.2019.7310 31200356

[9] George S Zheng Y Kim R Yu T Dreyfus J Gayle JA Real world outcomes of immune-related adverse events (irAEs) among patients receiving immune checkpoint inhibitors (ICIs) in hospital settings. Ann Oncol (2019) 30:v475–532. 10.1093/annonc/mdz253.109

[10] US Department of Health and Human Services Common Terminology Criteria for Adverse Events (CTCAE) Version 5.0. November 27, 2017.

[11] Putter H van Houwelingen HC Understanding Landmarking and Its Relation with Time-Dependent Cox Regression. Stat Biosci (2017) 9:489–503. 10.1007/s12561-016-9157-9 29225713 PMC5711994

[12] Cortellini A Chiari R Ricciuti B Metro G Perrone F Tiseo M Correlations between the immune-related adverse events spectrum and efficacy of anti-PD1 immunotherapy in NSCLC patients. Clin Lung Cancer (2019) 20:237–47 e1. 10.1016/j.cllc.2019.02.006 30885550

[13] Freeman-Keller M Kim Y Cronin H Richards A Gibney G Weber JS Nivolumab in resected and unresectable metastatic melanoma: Characteristics of immune-related adverse events and association with outcomes. Clin Cancer Res (2016) 22:886–94. 10.1158/1078-0432.CCR-15-1136 PMC4755809 26446948

[14] Fukihara J Sakamoto K Koyama J Ito T Iwano S Morise M Prognostic impact and risk factors of immune-related pneumonitis in patients with Non-Small-Cell Lung Cancer who received programmed death 1 inhibitors. Clin Lung Cancer (2019) 20(6):442–50. 10.1016/j.cllc.2019.07.006 31446020

[15] Sato K Correlation between immune-related adverse events and efficacy in non-small cell lung cancer treated with nivolumab. Lung Cancer (2018) 115:71–4. 10.1016/j.lungcan.2017.11.019 29290265

[16] Teraoka S Fujimoto D Morimoto T Kawachi H Ito M Sato Y Early Immune-Related Adverse Events and Association with Outcome in Advanced Non-Small Cell Lung Cancer Patients Treated with Nivolumab: A Prospective Cohort Study. J Thorac Oncol (2017) 12:1798–805. 10.1016/j.jtho.2017.08.022 28939128

[17] MenziesAMJohnsonDBRamanujamSAtkinsonVGWongANMParkJJ Anti-PD-1 therapy in patients with advanced melanoma and preexisting autoimmune disorders or major toxicity with ipilimumab. Ann Oncol (2017) 28:368–76. 10.1093/annonc/mdw443 27687304

[18] Danlos FX Voisin AL Dyevre V Michot JM Routier E Taillade L Safety and efficacy of anti-programmed death 1 antibodies in patients with cancer and pre-existing autoimmune or inflammatory disease. Eur J Cancer (2018) 91:21–9. 10.1016/j.ejca.2017.12.008 29331748

[19] Ricciuti B Dahlberg SE Adeni A Sholl LM Nishino M Awad MM Immune Checkpoint Inhibitor outcomes for patients with Non-Small-Cell Lung Cancer Receiving Baselin Corticosteroids for Palliative versus Nonpaaliative Indications. J Clin Oncol (2019) 37(22):1927–34. 10.1200/JCO.19.00189 31206316

[20] Martins F Sofiya L Sykiotis GP Lamine F Maillard M Fraga M Adverse effects of immune-checkpoint inhibitors: epidemiology, management and surveillance. Nat Rev Clin Oncol (2019) 16:563–80. 10.1038/s41571-019-0218-0 31092901

[21] Naidoo J Wang X Woo KM Iyriboz T Halpenny D Cunningham J Pneumonitis in Patients Treated With Anti-Programmed Death-1/Programmed Death Ligand 1 Therapy. J Clin Oncol (2017) 35:709–17. 10.1200/JCO.2016.68.2005 PMC5559901 27646942

[22] Cadranel J Canellas A Matton L Darrason M Parrot A Naccache JM Pulmonary complications of immune checkpoint inhibitors in patients with nonsmall cell lung cancer. Eur Respir Rev (2019) 28:190058. 10.1183/16000617.0058-2019 31597674 PMC9488121

[23] Lo JA Fisher DE Flaherty KT Prognostic Significance of Cutaneous Adverse Events Associated With Pembrolizumab Therapy. JAMA Oncol (2015) 1:1340–1. 10.1001/jamaoncol.2015.2274 PMC4679487 26270186

[24] Ma Y Pitt JM Li Q Yang H The renaissance of anti-neoplastic immunity from tumor cell demise. Immunol Rev (2017) 280:194–206. 10.1111/imr.12586 29027231

[25] Seliger B Combinatorial Approaches With Checkpoint Inhibitors to Enhance Anti-Tumor Immunity. Front Immunol (2019) 10:999. 10.3389/fimmu.2019.00999 31178856 PMC6538766

[26] FDA Keytruda Prescribing Information (2014). Available at: https://www.accessdata.fda.gov/drugsatfda_docs/label/2018/125514s041lbl.pdf (Accessed November 2, 2020).

